# Supplementary material for: Type 2 Diabetes Mellitus- and Complication-Related Risk of Nontuberculous Mycobacterial Disease in a South Korean Cohort
Source: Microbiol Spectr. 2023 Mar 28;11(2):e04511-22. doi: 10.1128/spectrum.04511-22 (PMC10100663; doi:10.1128/spectrum.04511-22)
Supplement: Supplemental file 1 — Tables S1 to S3. Download spectrum.04511-22-s0001.pdf, PDF file, 0.1 MB [file spectrum.04511-22-s0001.pdf]

**Supplementary Table 1.** Results of univariate and multivariable analyses of the associations between covariates and risk of nontuberculous mycobacterial disease

| Characteristics | Univariate analysis | | | Multivariate analysis | | |
|---|---|---|---|---|---|---|
| | Hazard ratio | 95% CI | *P*-value | Hazard ratio | 95% CI | *P*-value |
| Age, years | | | | | | |
| ≤39 | 1.00 | | | 1.00 | | |
| 40–49 | 0.97 | 0.74–1.23 | 0.843 | 0.94 | 0.72–1.23 | 0.640 |
| 50–59 | 1.48 | 1.16–1.89 | 0.002 | 1.31 | 1.02–1.67 | 0.033 |
| 60–69 | 2.43 | 1.91–3.09 | <0.001 | 1.94 | 1.52–2.48 | <0.001 |
| ≥70 | 2.83 | 2.20–3.63 | <0.001 | 2.14 | 1.65–2.77 | <0.001 |
| Sex | | | | | | |
| Male | 1.00 | | | | | |
| Female | 1.40 | 1.25–1.56 | <0.001 | 1.14 | 0.98–1.34 | 0.099 |
| Smoking status | | | | | | |
| Current or ever smoker | 0.70 | 0.62–0.79 | <0.001 | 0.85 | 0.72–1.00 | 0.054 |
| Comorbidity | | | | | | |
| Charlson Comorbidity Index | | | | | | |
| 0 | 1.00 | | | 1.00 | | |
| 1 | 1.83 | 1.58–2.10 | <0.001 | 1.44 | 1.24–1.67 | <0.001 |
| ≥2 | 2.20 | 1.92–2.51 | <0.001 | 1.33 | 1.12–1.58 | <0.001 |
| Bronchiectasis | 10.54 | 8.34–13.31 | <0.001 | 5.60 | 4.28–7.32 | <0.001 |
| COPD | 4.25 | 3.48–5.20 | <0.001 | 1.98 | 1.56–2.51 | <0.001 |
| Chronic kidney disease | 2.69 | 1.62–4.47 | <0.001 | 2.15 | 1.28–3.61 | 0.004 |
| Asthma | 2.29 | 1.99–2.64 | <0.001 | 1.40 | 1.19–1.65 | <0.001 |
| Malignancy | 2.19 | 1.72–2.80 | <0.001 | 1.57 | 1.22–2.03 | 0.001 |
| Dyslipidemia | 1.22 | 1.07–1.39 | 0.003 | 0.91 | 0.79–1.05 | 0.182 |
| Hypertension | 1.07 | 0.95–1.20 | 0.289 | | | |
| Health insurance type | | | | | | |
| Employee health insurance | 1.00 | | | | | |
| Self-employed health insurance | 1.06 | 0.94–1.19 | 0.330 | | | |
| Medical aid | 1.25 | 0.95–1.63 | 0.109 | | | |

**Supplementary Table 2.** Results of univariate and multivariable analyses of nontuberculous mycobacterial disease by type 2 diabetes mellitus and number of diabetes-related complications in 76,151 participants diagnosed with diabetes before 2007

| Settings | Total numbers | Matched pair | NTM-naïve T2DM cohort | | | NTM-naïve matched cohort | | | Crude HR (95% CI) | *P*-value | Adjusted HR (95% CI)* | *P*-value |
|---|---|---|---|---|---|---|---|---|---|---|---|---|
| | | | NTM disease | Person-year | Incidence (95% CI) | NTM disease | Person-year | Incidence (95% CI) | | | | |
| T2DM *vs*. no T2DM | 152,302 | 76,151 | 344 | 874,848.7 | 39.32 (35.28–43.70) | 272 | 891,701.2 | 30.50 (26.99–34.35) | 1.29 (1.10–1.52) | 0.002 | 1.07 (0.89–1.28) | 0.463 |
| T2DM with ≤1 complication *vs*. no T2DM | 114,740 | 57,370 | 242 | 676,146.8 | 35.79 (31.42–40.60) | 219 | 686,600.1 | 31.90 (27.81–36.41) | 1.13 (0.94–1.35) | 0.204 | 0.97 (0.72–1.19) | 0.759 |
| T2DM with ≥2 complications *vs*. no T2DM | 37,562 | 18,781 | 102 | 198,701.9 | 51.33 (41.86–62.31) | 53 | 205,101.1 | 25.84 (19.36–33.80) | 2.00 (1.43–2.79) | <0.001 | 1.68 (1.18–2.38) | 0.004 |

**Supplementary Table 3.** Results of univariate and multivariable analyses of nontuberculous mycobacterial disease by type 2 diabetes mellitus and number of diabetes-related complications in 115,067 participants diagnosed with diabetes after 2007

| Settings | Total numbers | Matched pair | NTM-naïve T2DM cohort | | | NTM-naïve matched cohort | | | Crude HR (95% CI) | *P*-value | Adjusted HR (95% CI)* | *P*-value |
|---|---|---|---|---|---|---|---|---|---|---|---|---|
| | | | NTM disease | Person-year | Incidence (95% CI) | NTM disease | Person-year | Incidence (95% CI) | | | | |
| T2DM *vs*. no T2DM | 230,134 | 115,067 | 365 | 752,053.4 | 48.53 (43.68–53.78) | 274 | 763,971.6 | 35.87 (31.74–40.37) | 1.35 (1.16–1.58) | <0.001 | 1.14 (0.97–1.34) | 0.113 |
| T2DM with ≤1 complication *vs*. no T2DM | 162,886 | 81,443 | 232 | 538,873.2 | 43.05 (37.69–48.96) | 177 | 545,353.1 | 32.46 (27.85–37.61) | 1.33 (1.09–1.61) | 0.005 | 1.19 (0.97–1.45) | 0.093 |
| T2DM with ≥2 complications *vs*. no T2DM | 67,248 | 33,624 | 133 | 213,180.2 | 62.39 (52.24–73.94) | 97 | 218,618.5 | 44.37 (35.98–54.13) | 1.41 (1.01–1.83) | 0.011 | 1.09 (0.83–1.43) | 0.521 |
